# Case report: A huge retroperitoneal solitary fibrous tumor closely related to the external iliac vessels misdiagnosed as an ovarian tumor

**DOI:** 10.3389/fmed.2024.1383961

**Published:** 2024-07-04

**Authors:** Danni Zhang, Li Wang, Lili Zhang, Shuzhi Yao, Juntong Wu, Song Han

**Affiliations:** ^1^Department of Obstetrics and Gynecology, The 964th Hospital, Changchun, Jilin, China; ^2^College of Chinese Medicine, Changchun University of Chinese Medicine, Changchun, Jilin, China; ^3^Department of Surgery, The 964th Hospital, Changchun, Jilin, China; ^4^Pathology Department, The 964th Hospital, Changchun, Jilin, China

**Keywords:** solitary fibrous tumor, surgical treatment, external iliac blood vessels, vascular replacement, ovarian tumors

## Abstract

**Background:**

Solitary fibrous tumor (SFT) is a rare soft tissue tumor originating from mesenchymal cells. Thus far, there have been no reported cases of SFT closely related to the iliac vessels.

**Case presentation:**

An elderly woman was found to have had a lower abdominal mass for more than 20 years. The enhanced computerized tomography (CT) showed a progressively enhanced hypervascular mass. The external iliac blood vessels were closely related to the mass, which was misdiagnosed as an ovarian tumor. During laparotomy, the external iliac vein was seen to penetrate the tumor, and the external iliac artery was seen to penetrate the tumor capsule. The retroperitoneal tumor was diagnosed during the operation. The surgical plan of complete tumor resection, severing of the external iliac arteries and veins, and blood vessel replacement was implemented. Pathological immunohistochemistry showed positive results for STAT6 and CD34, confirming the diagnosis of giant retroperitoneal SFT. The risk is classified as high and requires long-term follow-up. There has been no local recurrence or distant metastasis almost 1 year after surgery.

**Conclusion:**

The incidence of giant retroperitoneal SFT is rare, and the diagnosis can be confirmed through preoperative imaging examination and pathological examination. If the SFT capsule is intact, there is a chance of surgical resection. For SFTs that are penetrated by the iliac blood vessels, adequate preparation must be made before the surgery is performed. Removing the tumor and the iliac blood vessels at the corresponding site and then replacing it with artificial blood vessels is a feasible method with less risk of bleeding. In this case, imaging showed a progressively enhancing hypervascular mass in the lower abdomen, which was related to blood vessels. Preoperative biopsy and pathological testing can confirm the diagnosis. Neoadjuvant therapy or interventional therapy before surgery can shrink the tumor, making the surgical procedure relatively easy with less risk of bleeding.

## 1 Introduction

Solitary fibrous tumor (SFT) is a kind of fibroblastic tumor with extremely low incidence and clinical rareness (1). It was first described in pleural tumors by Klemperer and Rabin in 1931 (2), and the first extrathoracic SFT was reported in 1991 (3). This case reports a female patient who had a huge mass in her lower abdomen for more than 20 years and was misdiagnosed as an ovarian tumor. Based on the intraoperative findings and pathological results, she was finally diagnosed with SFT.

## 2 Case presentation

**Figure 1:**
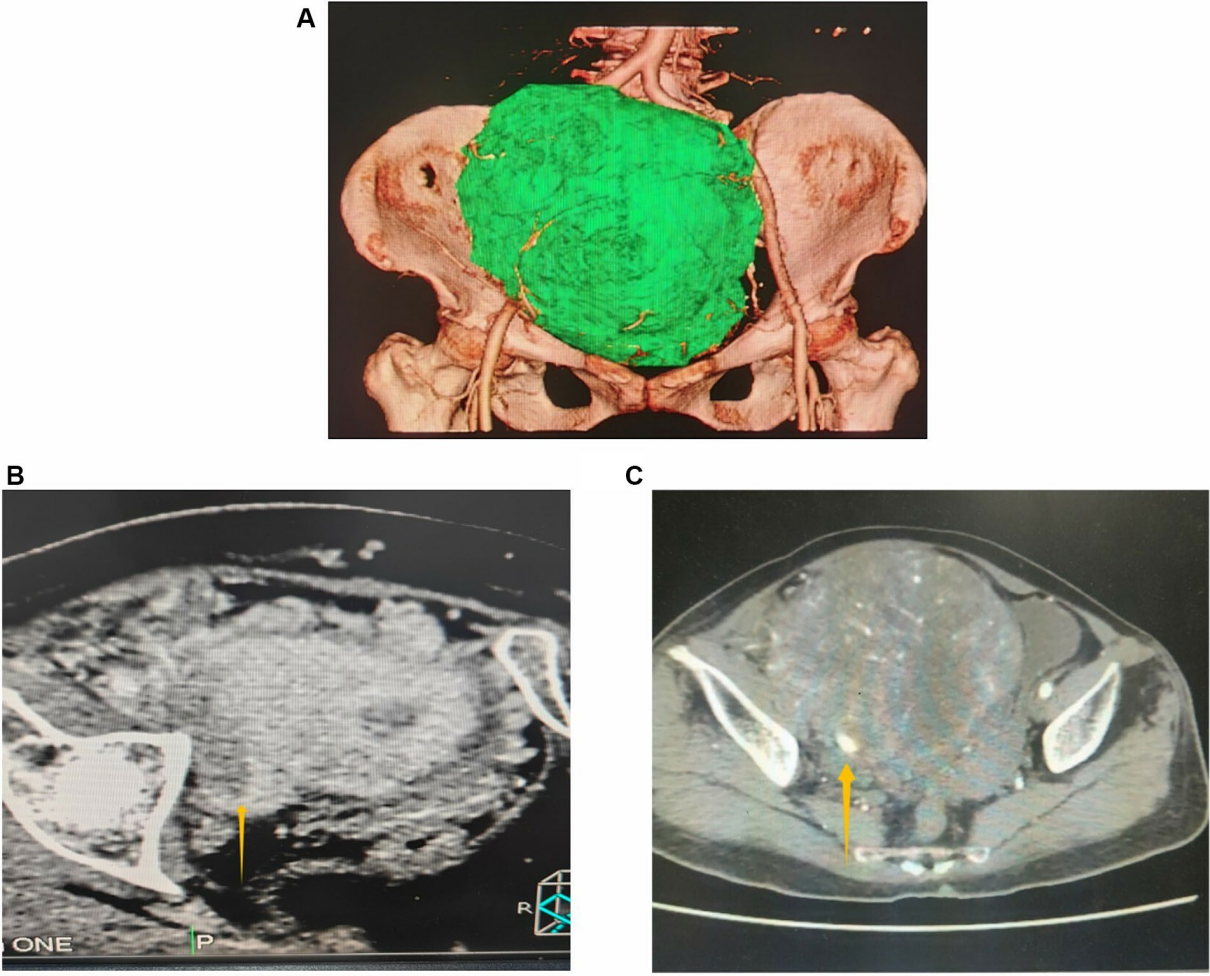
**(A)** Three-dimensional imaging based on CT scan shows a huge abdominal mass; **(B,C)** contrast agent-enhanced CT shows the cross-sectional view of the mass. The arrow **(B)** points to the external iliac vein; the arrow **(C)** points to the external iliac artery.

The patient is a 64-year-old woman who has had an abdominal mass for more than 20 years. She had no other symptoms except that the right lower limb was significantly thicker than the left lower limb. There was a history of hypertension and diabetes, and there was no history of cancer in the family. Abdominal physical examination revealed a mass of approximately 15 cm at the position below the umbilicus. It was on the right side, with poor mobility. The right lower limb was swollen. The right calf circumference was 33 cm, and the left calf circumference was 29 cm. The tumor markers, such as carcinoembryonic antigen, alpha-fetoprotein, cancer antigen (CA) 125, and CA199, were all negative. A color Doppler ultrasound of the blood vessels of the lower limbs showed a substantial heterogeneous echo measuring 14.8cmX10.6cmX16.7 cm at the original location of the right iliac vein. The blood flow was abundant, and the iliac vein could not be displayed. A CT examination showed a huge soft tissue density mass in the abdominal cavity measuring approximately 17cmX13cmX14cm (Figure 1A). Changes in lobulation and uneven internal density were seen. Most CT values were approximately 44Hu. Strip-like calcifications could be seen in the center, and patchy lower-density areas could be seen. Capsules were found around the lesions. After the injection of the contrast agent, arterial phase lesions showed obvious heterogeneous enhancement, and the solid components in the venous phase and the delayed phase were further filled and enhanced. The right external iliac artery and vein passed through the lesion (Figures 1B,C), along with multiple branch blood supply arteries originating from the right iliac blood vessels. The ureter and bladder were observed to be compressed. The uterus was reduced in size.

We initially diagnosed the mass as an ovarian tumor. Ultrasound and CT showed that the mass was closely related to the external iliac blood vessels. Before the operation, the patient and family were informed about the risks of the operation. The patient strongly requested surgery. An exploratory laparotomy was proposed. Vascular surgeons were informed, and in the event of vascular damage, doctors from two departments would complete the operation together.

**Figure 2:**
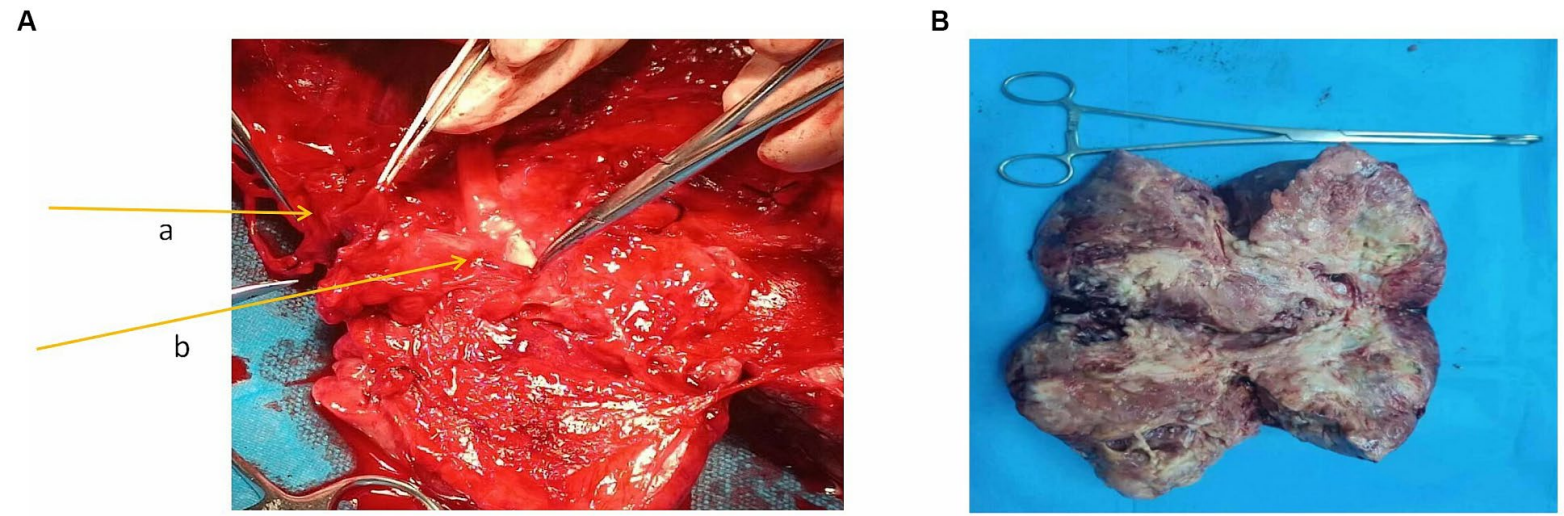
**(A)** External iliac blood vessels in the mass. The arrow a in the figure points to the external iliac vein; the arrow b points to the external iliac artery. **(B)** Macroscopic specimen.

During the operation, a mass with a diameter of approximately 17 cm was seen to be adhered and fixed to the right retroperitoneum, and a network of dilated, thin-walled blood vessels on its surface could be seen. The appearance of the uterus, bilateral ovaries, and fallopian tubes was normal and was pushed to the left side together with the bladder. Thus, the mass was diagnosed as a retroperitoneal tumor. After the obturator nerve was completely freed and the mass was lifted, a thick vascular pedicle formed by the right external iliac blood vessel was seen running through the capsule. After the incision of the capsule, it was found that the external iliac vein completely penetrated the tumor tissue and was not visible. The external iliac artery was located on the surface of the tumor and was covered with a capsule (Figure 2A). The external iliac vein could not be freed, and the external iliac artery had many branches. After consultation with the vascular surgeon, the external iliac arteries and veins were cut off while completely resecting the tumor. The artery was separated from the tumor by 8 cm, and the artery wall was not smooth. Tumor cell invasion could not be ruled out by the naked eye. The external iliac vein wall was severely infiltrated. A total of 4,000 units of low molecular weight heparin were administered to the patient, and external iliac artery and vein replacements were performed. An inferior vena cava filter was immediately placed after the operation. The frozen pathology during the operation was malignant, and a total hysterectomy with a double adnexectomy was performed. The patient recovered smoothly after the operation and was clinically cured.

## 3 Histopathology and immunochemical analysis

**Figure 3:**
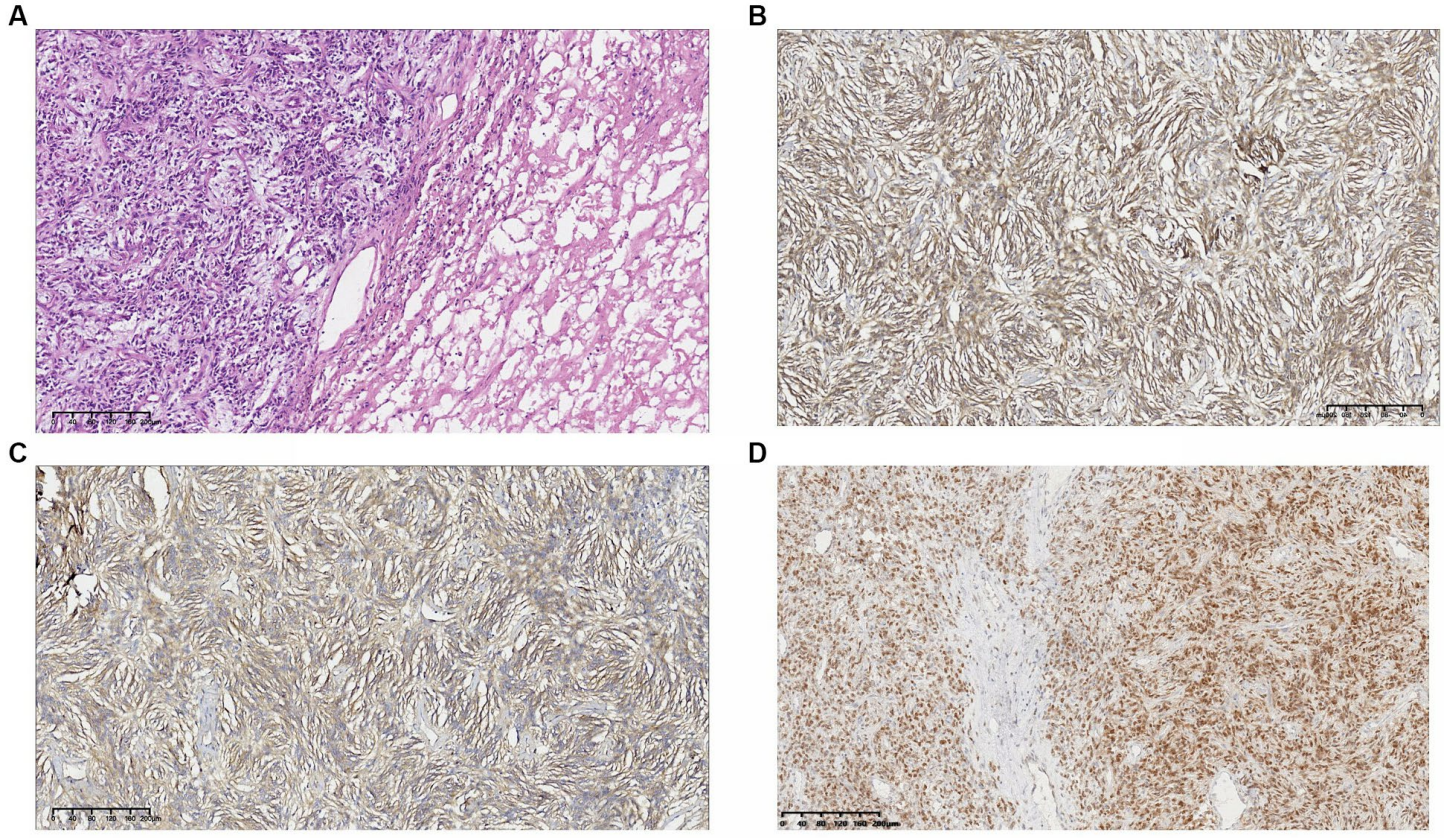
**(A)** Hematoxylin–eosin. 10X. The tumor consisted of densely packed and sparsely arranged areas of cells. The tumor cells were spindle-shaped or oval, with light eosinophilic cytoplasm, and partial nuclear atypia. Immunohistochemical result: **(B)** vimentin(+); **(C)** CD34(+); and **(D)** STAT6(+).

Macroscopic specimen, the tumor had been dissected with a size of 220mmX80mmX40mm (Figure 2B). The surface was covered with a fibrous pseudocapsule and was lobulated. The cut surface was yellowish-white, solid, and tough, and necrosis was seen in the center. Microscopic examination: the tumor consisted of densely packed and sparsely arranged areas of cells, accompanied by necrotic areas, and the stroma was rich in collagen fibers. Prominent branch blood vessels were visible, some with a “deer-antler” appearance. No infiltration of surrounding vessels and nerves was found. The tumor cells were spindle-shaped or oval, with small, darkly stained nuclei and moderate to light eosinophilic cytoplasm, partial nuclear atypia, and 4 mitotic figures per 10 high-power fields (HPFs) (Figure 3A). Immunohistochemistry results: The tumor was positive for vimentin, CD34, and STAT6 (Figures 3B–D) and negative for S-100, SYN, desmin, CK, CD56, Ki-67, and HMB-45. The above characteristics are consistent with the diagnosis of SFT.

**Figure 4:**
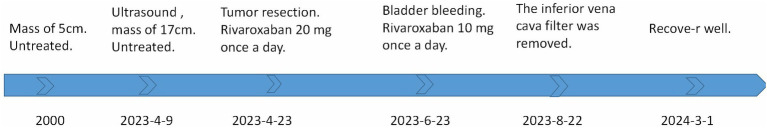
Timeline of the disease course.

The patient had good compliance and took the oral anticoagulant rivaroxaban (20 mg) once a day after surgery. Complications of bladder bleeding occurred 2 months later. The dose of rivaroxaban was reduced to 10 mg once a day. The inferior vena cava filter was smoothly removed 4 months later. There has been no local recurrence or distant metastasis almost a year after the operation. The thickness of both lower limbs is basically the same. The patient recovers well and is very satisfied (Figure 4).

## 4 Discussion

The key point in this case is the correct diagnosis. The incidence of SFT is 0.1 per 100,000 person-years (4). It is clinically rare and, thus, difficult to diagnose in this case. Retroperitoneal SFT tumors are usually large, and patients often suffer from abdominal discomfort, sacrococcygeal pain, or defecation disorders due to tumor compression. A minority of patients have paraneoplastic syndromes. The most common symptoms are non-islet cell hypoglycemia, hypertrophic osteoarthropathy, or digital clubbing (5). Because the clinical symptoms of SFT are non-specific, imaging examinations are still the main method for diagnosing SFT (6). The CT scan showed a clear, occasionally lobulated mass with a density equal to that of skeletal muscle. MRI indicated that larger or invasive cases show increased heterogeneity with intensity on T1-weighted images and variable hypointensity to hyperintensity on T2-weighted images (1). CT or imaging features of SFTs include highly vascular enhancement, possibly due to high fiber content, showing progressive enhancement in the arterial and venous phases that becomes apparent on delayed imaging (7). This patient has had a disease course of more than 20 years and presents with a huge mass. Preoperative CT has revealed a highly heterogeneous blood vessel mass. The ipsilateral lower limb was thickened, accompanied by the symptoms of obstruction of blood circulation. SFT was not considered at first due to the doctor’s lack of experience. The tumor’s proximity to the ovary became another factor leading to the misdiagnosis of an ovarian tumor.

SFT tumor cells usually consist of uniformly ovoid to spindle-shaped cells arranged in a patternless manner in a variable collagen matrix. There are scattered large, dilated, thin-walled, branching “antler” vessels (8). Dedifferentiation of SFTs is characterized by an abrupt transformation into low-grade or high-grade sarcoma with adjacent conventional SFTs (4). Typical SFT manifestations on immunohistochemistry are diffuse expression of CD34, CD99, and BCL2. SFT is thought to be caused by the fusion of the transcription repressor NAB2 and the transcription factor STAT6 on chromosome 12. Immunohistochemistry STAT6 is a sensitive and specific surrogate for the fusion gene and is also expressed in malignant cases. Although dedifferentiated SFTs have been shown to retain the NAB2-STAT6 fusion gene, they have reduced or lost expression of the chimeric protein, resulting in the absence of STAT6 in immunohistochemistry. This emphasizes the importance of molecular diagnosis as a standard treatment for patients with soft tissue sarcomas (9). This patient was diagnosed with SFT based on histopathology and positive immunohistochemistry for STAT6 and CD34. Unfortunately, the mass in this case was huge, and an easy-to-operate biopsy and pathological immunohistochemical examination were not performed, which became another factor in the misdiagnosis. Nishino’s research shows that when a tumor compresses an adjacent non-originating plastic organ (such as the gastrointestinal tract and inferior vena cava), the organ becomes crescent-shaped; conversely, when part of the organ appears to be embedded in the tumor, the tumor is likely to originate from that organ (10). The CT examination and intraoperative findings of this patient showed that the external iliac vein was embedded in the mass. Currently, the possibility that the tumor originated from the iliac blood vessels cannot be ruled out, yet there is insufficient evidence to prove this.

Radical resection is still the first-line treatment for retroperitoneal SFT (11). The intraoperative challenge in this case was whether to isolate the iliac vessels and then resect the tumor or to directly cut off the iliac vessels to completely resect the tumor. Additionally, the choice between using the isolated autologous iliac artery, taking the autologous great saphenous vein, or using artificial blood vessels added to the complexity of this case. Patient prognosis is related to the completeness of tumor resection rather than histological grade (12). However, since SFT is a highly vascularized tumor, surgery becomes difficult, and complete resection is not easy. Inadequate mass resection, aneurysms, uncontrollable massive bleeding, and even life-threatening risks may occur (13, 14). Although patients have undergone vascular embolization before surgery, massive bleeding during surgery is still inevitable, and some methods need to be taken to control bleeding (15). In order to improve the survival rate of patients, even in patients with SFT of major vessels (such as the superior vena cava or inferior vena cava), tumors should be completely resected (16–21). The characteristics of this case were that the mass was huge and was located deep in the obturator fossa. The external iliac artery and vein penetrated the tumor, with the external iliac vein especially penetrating the tumor and could not be separated. The external iliac artery penetrated the tumor capsule and was closely related. In order to completely remove the tumor and reduce the risk of intraoperative bleeding, doctors from two departments decided to cut off the external iliac arteries and veins and then completely remove the tumor. After the external iliac arteries and veins were freed, the naked eye could not rule out the possibility of tumor cell invasion in the blood vessel wall. Considering the thin wall and small diameter of the great saphenous vein, we decided to perform artificial blood vessel replacement and insert a filter into the inferior vena cava. Some scholars have reported that neoadjuvant radiotherapy or interventional techniques can be used to shrink tumors, improve symptoms, and facilitate surgical resection. Targeted radiotherapy with 56–60 Gy can reduce the volume of pelvic or thoracic tumors by up to 60%, and adjuvant radiotherapy does not increase the risk of perioperative complications (22). External iliac artery embolization may affect the blood supply to the lower limbs, while internal iliac artery embolization is a more ideal choice. Unfortunately, the above measures were not implemented before surgery in this case to reduce the risk. If the preoperative diagnosis was confirmed and neoadjuvant radiotherapy or interventional techniques were performed to reduce the tumor volume, it is possible to separate and preserve the external iliac artery. However, both the external and internal iliac arteries were the blood supply arteries for the tumor, and there was also a possibility of significant bleeding after the internal iliac artery embolization. In the excised tumor specimen, the external iliac vein wall was severely infiltrated, and the artery wall was not smooth. If interventional embolization of the internal iliac artery had been performed, it would have been possible to separate and preserve the external iliac artery. Tumor tissue residue could not be ruled out. It is more accurate to predict the risk of tumor metastasis using the Demicco risk score (23). The patient’s age of 64 years old was scored as 1 point; mitosis ≥4/10HP was scored as 2 points; tumor size ≥15 cm was scored as 3 points; tumor necrosis ≥10% was scored as 1 point; and the total score was 7 points. This score indicates a high risk, and the patient should be followed up for a long time after surgery.

## 5 Conclusion

SFT imaging shows an enhanced hypervascular mass, which should be taken seriously. A puncture biopsy can increase the accuracy of the preoperative diagnosis, but molecular examination is more reliable. The risk of surgery for tumors closely related to iliac vessels increases. Under difficult circumstances, cutting off the iliac blood vessels to completely remove the tumor and artificial iliac blood vessel replacement are radical treatments worth considering.

## Data availability statement

The original contributions presented in the study are included in the article/supplementary material, further inquiries can be directed to the corresponding author.

## Ethics statement

The studies involving humans were approved by Ethics Committee of 964 Hospital. The studies were conducted in accordance with the local legislation and institutional requirements. The participants provided their written informed consent to participate in this study. Written informed consent was obtained from the individual (s) for the publication of any potentially identifiable images or data included in this article.

## Author contributions

DZ: Writing – original draft. LW: Writing – review & editing. LZ: Writing – review & editing. SY: Writing – original draft, Writing – review & editing, Methodology, Conceptualization. JW: Writing – original draft. SH: Writing – review & editing.
